# 

**Published:** 1896-02

**Authors:** 


					﻿Vol. XVI.
FEBRUARY, 1896.
NO. 2.
THE
Pomœopathic physician,
A Monthly Journal of Homoeopathic Materia Medica
and Clinical Medicine.
“ He is a freeman whom truth makes free, and all are slaves beside.”
"Seek the Truth: come whence it may, cost what it will."
EDITED AND PUBLISHED BY
WALTER M. JAMES, M. D.
PHILADELPHIA :
TTo. 1231 LOCVST STREET.
LONDON:
ALFRED HEATH <fc CO., Sole Agents for Great Britain,
114 Ebury Street, Eaton Square.
Yearly Subscription, $2.SO, invariably in advance. Single numbers t 25 els.
Entered it the Philadelphia Poit-OIBce u Second-Clue Mail Matter.
				

